# Utilization of Partograph and its associated factors among midwives working in public health institutions, Addis Ababa City Administration,Ethiopia,2017

**DOI:** 10.1186/s12884-020-2734-4

**Published:** 2020-01-21

**Authors:** Azeb Abrham Hagos, Eshetu Cherinet Teka, Genet Degu

**Affiliations:** 1 Addis Ketema Sub City Health Office, Family Health Case Team, Addis Ababa, Ethiopia; 2 Ministry of Health-Ethiopia, National Health Professionals Competency Assessment and licensure Directorate, Addis Ababa, Ethiopia; 3 Health Science College, Midwifery Department, Debre Markos University, Debre Markos, Ethiopia

**Keywords:** Utilization, Partograph, Public health facilities, Addis Ababa, Ethiopia

## Abstract

**Background:**

Partograph is cost effective and affordable tool designed to provide a continuous pictorial overview and labour progress used to prevent prolonged and obstructed labour. It consists of key information about progress of labour, fetal condition and maternal condition. Its role is to improve outcomes and predict the progress of labour. The aim of this study was to assess utilization of partograph and its predictors among midwives working in public health facilities, Addis Ababa city administration, Ethiopia, 2017.

**Methods:**

An institution based cross-sectional study design was conducted in Addis Ababa, Ethiopia from 15/10/2017–15/12/2017.Simple random sampling under multistage sampling technique was applied to select a total of 605 midwives working in maternity unit of selected public health facilities. Data were collected using structured self-administered questionnaire. Checklist based direct observations were made to all midwife participants to determine the actual practical use of partograph. Data first entered in to EpiInfo version 3.5.1 and transported to SPSS Version 21.Descriptive statistics such as frequency, percentage, mean, and median were calculated. Biviriate and multivariable logistic regression analysis were applied. Any personal identification of the study participants was not recorded during data collection to ensure confidentiality of information.

**Results:**

In this study, the utilization of partograph was 409(69%) out of 594 study participants. Being mentored(AOR = 3.1; 95% CI: 1.7, 5.3),received training (AOR = 2.4; 95% CI:1.5,3.6),being knowledgeable about partograph (AOR = 1.6; 95% CI: 1.1, 2.5), health center workers(AOR = 12.6; 95% CI:5.1,31.6),supportive supervision 4 times per year (AOR = 18.6; 95% CI: 6.6,25),supportive supervision twice per a year (AOR = 4.7; 95% CI: 1.9, 11.3),supportive supervision once per year (AOR =3.8;95% CI:1.7,8.8) were positive predictors of partograph utilization. Two midwives per shift (AOR = 0.101; 95% CI: 0.05, 0.65), and 4 per shift (AOR = 0.105, 95% CI: 0.03, 0.40) were protective predictors of partograph utilization.

**Conclusions:**

More than half of the respondents utilized partograph. All public health institutions avail partograph in their laboring room but didn’t utilize it according to WHO recommended standard. Working facility, supportive supervision, mentoring, training on partograph, number of midwives working per shift, and knowledge were factors affecting partograph utilization. Encouraging interventions are recommended to the response of the above significantly associated factors.

## Background

Globally, around 303,000 maternal deaths occurred in the year 2015 during and following pregnancy or childbirth. Of all the deaths, 99% were in developing countries in which 546 per 100,000 live births (66%) of them occurred only in Sub-Saharan Africa [1]. According to the 2000 Ethiopia Demographic and Health Survey, maternal mortality ratio was 871per 100,000 live births in Ethiopia. Though it was slightly declined to 673 in 2005, a slight increment was observed to 676 in 2011 [2]. Maternal mortality was still high in 2016 accounted to 412 per 100,000 live births [3].

Most of the time, maternal deaths and complications are the results of obstructed and prolonged labor. Prolonged labor is a leading cause of death among mothers and newborns in the developing world. If the labour does not progress normally, a woman may experience serious complications such as obstructed labor, dehydration, exhaustion, or rupture of the uterus. It may also contribute to maternal infection or hemorrhage and to neonatal infection. This can be prevented by accessing skilled delivery services such as plotting partograph during the progress of labour [4, 5].

Partograph is cost effective and affordable tool designed to provide a continuous pictorial overview of labour progress used to prevent prolonged and obstructed labour. It consists of key information about progress of labour, fetal condition and maternal condition. Its role is to improve outcomes labour; predict the progress of labour; leads to an on time decision and proven intervention [6].

World Health Organization (WHO) recommends the partograph to be used for monitoring all laboring mothers. It is still not broadly used in the developing world especially in Africa due to different factors such as lack of human resources,time pressure, stock-outs of partograph paper, inadequate monitoring of maternal and fetal key indicators [7]. In Nigeria, 70.8% of obstetric care givers were well aware and had good general knowledge of the partograph but far below expectation. They also lacked detailed knowledge of the components [8].

In Ethiopia, midwives in labour and delivery room are not using partograph to all mothers continuously to follow the labour process [9]. Similarly, different health facilities not utilized partograph due to different determinant factors such as: time of admission, nature of membrane during admission, knowledge, training, attitude [10],Sex, lack of institutional policy to utilize partograph and number of health professionals per shift [11].

Assessing midwives’ practical utilization of partograph and its determinants has great value to design appropriate intervention strategies to provide quality maternity care. Therefore, the aim of this study was to assess utilization of partograph among midwives working in public health institutions of Addis Ababa City administration.

## Methods

### Study setting and period

This study was conducted in public health institutions from October 15, 2017 to December 15, 2017 in Addis Ababa which is the capital city of Ethiopia with a population over 3 million (3,433,999) people. It is divided in to ten sub-cities and 116Kebeles (lowest administrative units in Ethiopia). This city is located at 9° 1′ 48″ north and 38° 44′ 24″east and the total land area is 54,000 ha [2]. There are 11 public hospitals of which 6 are under the administrative unit of Addis Ababa Regional Health Bureau (AARHB) and the rest 5 are under Federal Ministry of Health. Furthermore, the city has 103 public health centers. The total number of midwives working in public health institutions was 1017(Addis Ababa Food Medicine and Health care Administration authority Annual review meeting Addis Ababa,unpublished. 2017/2018).

### Study design and population

**Figure 1:**
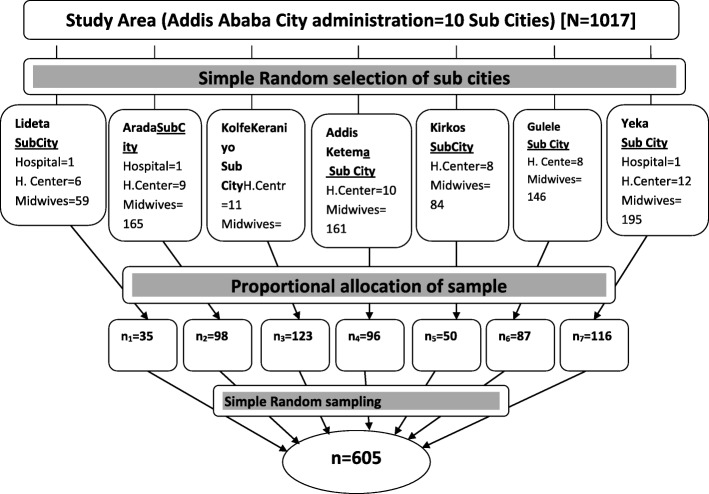
Sampling Procedure: Schematic presentation of the sampling procedure, Addis Ababa, Ethiopia, 2017

An institution based cross-sectional study design was conducted to assess utilization of partograph and associated factors. Multi stage sampling technique was employed to recruit participants. Firstly, Addis Ababa City Administration was selected as a study area. Secondly, seven of the ten sub cities were selected using simple random sampling technique. They comprised of 64 health centers and 3 hospitals and sample size to each selected sub city was determined proportionally. Thirdly, all public hospitals and health centers in selected sub cities were included and midwives working in each health facility were determined proportionally (Fig. 1).

Finally, those who were working a minimum of six months in maternity unit were selected randomly as a sample. Sample size was determined using a single population proportion formula *n* = (Za/2)^2^P (1−*р*)/ *d*^2^ assuming that n: the desired sample size, Z: significance level, P: proportion/prevalence, d: margin of error. We used 95% confidence level, 57.4% proportion of partograph utilization from the study done in Addis Ababa, Ethiopia in 2013 [12]. We corrected sample size by final correction formula (nf = n/1 + n/N) to be 275.By considering design effect 2and 10% non-response rate, the minimum sample size required was estimated to be 605 midwife participants (Degu G. and Tessema F. sampling method. Biostatistics lecture note for health science students. Ethiopia, January2005:181–182, (https://newonlinecourses.science.psu.edu).

### Data collection and quality assurance

Firstly, direct observation was undergone by data collectors to all midwives who were involved in the study to check whether they used and filled appropriately parameters of the partograph or not (Additional file 1: Annex 1). Observation was made till the end of delivery. Observation checklist was developed as a tool from the partograph chart for direct observation of study participants during the progress of labour. Secondly, data were collected using structured self-administered questionnaire about midwives’ partograph utilization and associated factors. It was developed from different literatures considering the research objectives. It consisted of socio-demographic characteristics, knowledge related questions, attitude related questions and practice related questions. Seven midwives as volunteer data collectors and three public health professionals as supervisors were recruited and trained. The questionnaire was prepared in English then translated to Amharic by an expert. The tool was retranslated back to English to maintain its consistency. Data collectors distributed the questionnaire to midwives who were selected as a sample after explaining the purpose of the study and obtaining written informed consent.

Collection boxes were provided in the maternity wards for depositing the completed questionnaires to maintain confidentiality. To maintain the consistency of the tool, questionnaire was back translated to English by language experts. The tool was pretested with 30(5% of study participants) midwives working in Bole Sub City Public Health Centers which were not part of the study. Then, data collection tools were amended and rearranged to accommodate the desired data for the study. Field supervisors rechecked clearness and completeness of the filled questionnaire immediately after collecting the data from the field level and during submission.

During direct observation, Midwives who had been recording at least fetal heart rate, cervical dilatation, uterine contraction, vital signs, amniotic fluid, and descent of fetal head completely and correctly in the partograph for all laboring mothers were considered as partograph utilizers. To estimate the overall knowledge about partograph, nine knowledge related questions about partograph were prepared and midwives who answered five and above were considered to have good level of knowledge unless said to have poor level of knowledge [13]. To estimate the overall attitude of respondents, six attitude related questions were prepared and each measured by using a 5-point Likert scale namely strongly agree, agree, uncertain, disagree and strongly disagree. Respondents who replied at least 3 questions (either strongly agree or agree) were considered to have positive attitudes while those who replied 3 or more disagree or strongly disagree were considered to have negative attitude towards partograph utilization [14].

### Data processing and statistical analysis

The data was checked visually for completeness then coded and entered into EpiInfo version 3.5.1 and exported to statistical package for social science (SPSS version 21). Descriptive analysis such as frequency, percentage, mean, and median were calculated for different variables and bivariate and multivariable analysis were performed to identify factors associated with utilization of partograph. Statistical level of significance was stated at *p* value < 0.05 to indicate the association of independent variable with dependent variable.

## Results

### Socio-demographic characteristics of study participants

Out of 605 study participants, 594 completed the questionnaire correctly making a response rate of 98.1%. Females midwives were 443(74.6%) while males were 151(25.4%). Then mean age of midwives was 25 years with a standard deviation of 0.8. Three fourth of the obstetric ward working midwives had less than 5 years of service. Those who had a service year of 5–10 years were 129 (21.7%) while those served more than 10 years were 14(2.4%) (Table 1).

**Table 1:** Socio-demographic characteristics of midwives in public health institutions of Addis Ababa City Administration, Ethiopia, 2017 (*N* = 594)

Variable	Number	Percent
Sex		
Male	151	25.4
Female	443	74.6
Age category		
20–24	195	32.8
25–29	291	49.0
30–34	75	12.6
35–39	22	3.7
> 40	11	1.9
Marital status		
single	356	59.9
Married	219	36.9
Divorced	13	2.2
Widowed	2	0.3
Separated	4	0.7
Service year		
< 5 years	451	75.9
5–10 years	129	21.7
> 10 years	14	2.4

### Knowledge of the study participants’ on partograph

The overall knowledge of midwives was511(86%,CI: 84.6, 87.4%).Nearly all (98%, of participants mentioned at least one component of the partograph (not all components). More than two third of them define what partograph means but Less than half of the participants knew the function of action line (Table 2).

**Table 2:** Knowledge of midwives about Partograph in Addis Ababa City Administration, Ethiopia, 2017 (*n* = 594)

Variable	Frequency	Percent
Over all Knowledge about partograph		
Yes	511	86
No	83	14
Mentioned all components of the partograph		
Yes	424	71
No	170	29
Mentioned at least one component of partograph (not all)		
Yes	582	98
No	12	2
Training on partograph		
Pre-service	318	53
In service	276	47
Describe function of action line on partograph		
Yes	251	42
No	343	58
The partograph will reduce maternal deaths		
Yes	523	88
No	71	12
The partograph will reduce new born deaths		
YES	517	87
No	77	13

### Utilization of Partograph

**Fig. 2:**
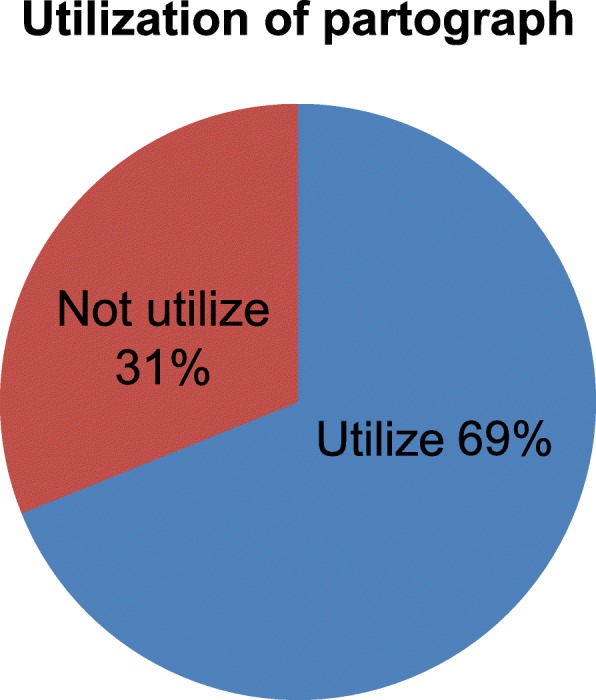
utilization of partograph while attending labour in selected health institutions of Addis Ababa city Administration, Ethiopia, 2017 (N = 594)

Direct observation was made to all 594 midwife Participants while attending the progress of labour. It indicated that 537(90%) participants recorded blood pressure while 409(69%) participants recorded amniotic fluid during the progress of labour. They recorded temperature, cervical dilatation and uterine contraction almost in a similar frequency that is 528(89%), 532(90%), 525(88%) respectively (Table 3). In this study, 409(69%, CI: 67.1, 70.9%) midwives utilized partograph during the progress of labour (Fig. 2).

**Table 3:** Parameters recorded by midwives in partographs while attending labour in Selected health institutions of Addis Ababa City Administration, Ethiopia, 2017 (*N* = 594)

Variable	Frequency	Percentage
Uterine contraction		
Yes	525	88
No	69	12
Cervical dilatation		
Yes	532	90
No	62	10
Fetal heart rate		
Yes	511	86
No	83	14
Blood pressure		
Yes	537	90
No	57	10
Temperature		
Yes	528	89
No	66	11
Amniotic fluid		
Yes	409	69
No	185	31

### Participant’s reason for not using the partograph

**Fig. 3:**
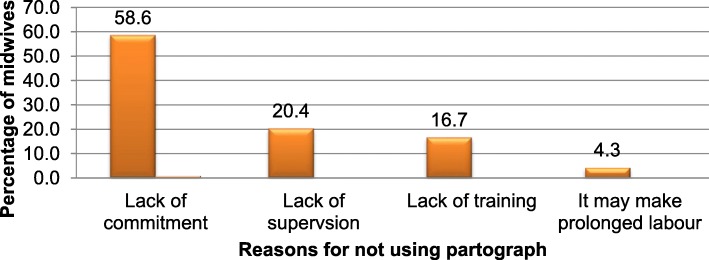
Reasons for not using the partograph properly in Addis Ababa City Administration public health facilities, Ethiopia, 2017 (*N* = 594)

According to this study, the main reasons that were reported against the effective use of partograph by the midwives were lack of commitment 348(58.6%), lack of supervision 121(20.4%), lack of training 99(16.7%) followed by attitudinal issue in which 26(4.3%) midwives perceived that using partograph may make prolonged labour (Fig. 3).

### Attitude of respondents towards partograph utilization

The attitude of the respondents’ was assessed and almost all 578(97.4%) of the respondents agreed that partograph is beneficial. Similarly, 580(97%) respondents strongly agreed that partograph is favorable as it alerts obstetric care givers of any deviation from normal and 575(97%) agreed that health care providers are able to identify problems and recognize complications early. On the other hand, 540(90.9%) strongly agreed that using partograph enables health care providers perform essential basic interventions and make referrals to appropriate levels of care (The overall attitude score of the respondents was 571(96.2%,CI:95.4,97.0) (Table 4).

**Table 4:** Respondents’ attitude on partograph utilization in public health institutions in Addis Ababa City Administration, Ethiopia; 2017 (*N* = 594)

Attitude statement	Favorable	Unfavorable
partograph is useful for attending labour	578(97.4%)	16(2.6%)
The partograph is very favorable as it alert skilled birth attendant any deviation from normal	580(97.0%)	14(3.0%)
By using a partograph midwives are able to identify problems, recognize complications early.	575(97.0%)	19(3.0%)
Midwives mandatory use a partograph on every laboring mother.	571(96.1%)	23(3.9%)
Using partograph enables midwives perform essential basic interventions and make referrals to appropriate levels of care when necessary	540(90.9%)	54(9.1%)
Using partograph is not beneficial as the estimate it gives is exaggerated	185(31.2%)	409(68.8%)
Over all attitude score	571(96.2%)	23(3.8%)

### Factors associated with partograph utilization

Bivariate logistic regression statistical test was done and the presence of any relationship between independent variable with partograph utilization as dependent variable among obstetric unit working midwives was examined. Factors like age of midwives, marital status, service year, knowledge on definition of partograph, attitude towards partograph, problem in using partograph didn’t reveal statistically significant association (*p* > 0.2) with utilization of partograph.

Those variables with *P* < 0.2 during bivariate logistic regression analysis were included under multivariable logistic regression model to control the possible confounder variables and identified the real predictor variables. But, only variables with *p* < 0.05 were considered to have a significant association with utilization of partograph. As a result, six variables namely mentor assign, training on partograph, knowledge; working facility, supportive supervision, and number of midwives work per shift were found to be factors associated with use of partograph.

The odds of partograph utilization for midwives mentored by an expert were 3 fold higher than those not mentored (AOR = 3.0; 95% CI: 1.8, 5.4). The odds of partograph utilization for midwives who were trained on partograph was 2 fold higher than those not took training (AOR = 2.4; 95% CI: 1.5, 3.7). The odds of partograph utilization for midwives who knew about partograph was 2 fold higher than those not knew (AOR =1.6; 95% CI: 1.1, 2.5).

The average delivery in a health center is 5 per a day (shift) and 11 in a hospital according to the data taken from the registration of health centers and hospitals included in this study by taking a one month data prior to the data collection period. The odds of partograph utilization for midwives working at health center was 13 fold higher than hospital workers (AOR = 12.7; 95% CI: 5.1, 31.6). The odds of partograph utilization for midwives supported once in a year by supportive supervision was 4 fold higher than those not supported (AOR = 18.6; 95% CI: 6.6, 25). The odds of partograph utilization for midwives supported by supportive supervision 2 times in a year was 5 fold higher than those not supported (AOR = 4.7; 95% CI:1.9, 11.3). The odds of partograph utilization for midwives supported once in a year by supportive supervision were 4 fold higher than those not supported (AOR = 3.8; 95% CI: 1.7, 8.8).

Those midwives working 2 per shift were 89.9% less likely to utilize partograph than midwives working 8 per shift (AOR = 0.101;95% Cl:0.05,0.65) Midwives working 4 per shift were 89.5% less likely to utilize partograph than midwives working 8 per shift (AOR = 0.105, 95% CI: 0.03,0.40) (Table 5).

**Table 5:** Factor associated with partograph utilization of midwives in public Health institutions of Addis Ababa City Administrations, Ethiopia, 2017. (*N* = 594)

Variables	Utilization of partograph		COR	AOR	Significance (*P*-value)
	Utilize No(%)	Not utilize No (%)			
Mentored					
Yes	263(62.5)	158(37.5)	3.3(2.1–5.1)	3(1.8–5.4)	0.00
No	146(84.4)	27(15.6)	1	1	
Trained					
Yes	156(56.5)	120(43.5)	3(2.1–4.3)	2.4(1.5–3.7)	0.00
No	253(79.6)	65(20.4)	1	1	
Knowledge					
Yes	310(60.6)	201(39.4)	1.9(1.8–2.7)	1.6(1.1–2.5)	0.03
No	62(74.7)	21(25.3)	1	1	
Working Facility					
Hospital	133(80.4)	35(19.6)	1	1	
Health Center	276(64.8)	150(35.2)	2.1(1.4–3.2)	12.7(5.1–31.6)	0.00
Supportive Supervision					0.00
4 times in a year	19(34.5)	36(65.5)	11.7(5.0–27.3)	18.6(6.6–25.0)	0.00
2 times in a year	82(60.7)	53(39.3)	4(1.9–8.0)	4.7(1.9–11.3)	0.01
1 times in a year	240(73.8)	85(26.2)	2.2(1.1–4.3)	3.8(1.7–8.8)	0.01
Not supervised	68(86.1)	11(14)	1	1	
Midwives Work Per Shift					0.005
2 midwives	195(69.4)	86(30.6)	0.4(0.2–0.7)	0.101(0.05–0.65)	.010
4 midwives	139(75.1)	46(24.9)	0.3(0.2–0.6)	0.105(0.03–0.40)	.001
6 midwives	54(65)	29(35)	0.5(0.2–0. 9)	0.5(0.20–1.30)	.139
8 mid wives	20(46.5)	23(53.5)	1	1	

## Discussion

This study was intended to determine magnitude and determinant factors of utilization of partograph among midwives of public health institutions of Addis Ababa. In this study, the utilization of partograph was 68.9%.

This magnitude is higher than the study conducted in Amhara region North Shoa Zone (40.2%) [15]. In Malawi, only 10% of the pantographs had correct and complete information in each parameter of all the three components [16]. In Ethiopia, Jimma University,only 19/274 (6.9%) of mothers monitored but only in 2(10.53%) had correct documentation and monitoring system [17]. Besides, in India Mahatma Gandi University, only15 (50%) of obstetric care givers use partograph to monitor women in labour [18]. This magnitude is lower than the study conducted in South Africa implying that over two thirds (79.4%; *n* = 54) of participants routinely used partograph [19]. The differences between these findings might be due to methodological differences such as differences in study participants, study area and time;differences in implementation strategy; policy and commitment towards using partograph routinely for each laboring mother. Negative attitude and lack of training might be barriers for the above utilization differences. The role of training for utilization of partograph is explained in Rwanda [20], Cameroon [21], Nigeria-Calabar [22], Ghana-Kumasi [23],Uganda [24],and Ethiopia-Bale Zone [25].

The utilization of partograph in this study was also 11.5% more higher than the study conducted in Addis Ababa 6 years ago(2013) which was 57.3% [12]. Clearly it can be said that lots of basic and refreshment trainings related to partograph were given to build the capacity of Obstetrics/gynecology workers. Besides, the difference in the data collection procedure, time gap leading to change in policy, strategy and improvement in implementation of the partograph.

This study showed that all parameters of partograph were not filled by all participants. Fetal heart rate; cervical dilation and uterine contraction were recorded by more than 85% but not monitored according to the standard till the end. This finding is consistent with the study in Amhara,Ethiopia [9]. This might be due to similarity in health care administration system since they are in the same country.

In this study, there were six independent variables namely Type of working facility, supportive supervision, mentoring, training on partograph, number of midwives working per shift, Knowledge on partograph were significantly associated with utilization of partograph. Four of the variables were supported by previous studies while supportive supervision and mentoring of midwives were identified as new additional explanatory variables.

According to this study, working in health center was 13 times more likely to utilize partograph as a tool guide than hospital workers. In Ethiopia, West Shoa zone, hospital workers were less likely to utilize partograph than working at primary health care unit [26]. Possible explanation for this might be midwives at health centers utilize partograph as a guide to take an action early to have adequate evidence even to refer to higher health institution. Moreover, they had less client load than hospitals. On the contrary, Hospital worker midwives might have over confidence than health center workers that lead them not to follow the labor progress by using partograph. The reason might be hospital worker midwives think they can easily manage disorders and complications at their own ground knowledge without wasting time by transporting the laboring mother to other health institution.

In this study, midwives who were trained on partograph were 2 times more likely to utilize partograph than those not took training. A similar finding was observed in Rwanda [20],Cameroon [21], Nigeria-Calabar [22],Ghana-Kumasi [23],Ethiopia-Bale Zone [25],and Uganda [24]. This might be due to the program of the Ministry of Health-Ethiopia aimed to train skilled birth attendants country wide in emergency Obstetric and newborn care.

According to this finding, midwives in health facilities Supervised 4 times in a year were 18 times more likely to utilize partograph than not supervised midwives. Midwives in health facilities Supervised 2 times in a year were 5 times more likely to utilize partograph than not supervised midwives. Those Midwives in health facilities Supervised once in a year were 4 times more likely to utilize partograph than those not supervised midwives. Midwives mentored by experts were 3 times more likely to utilize partograph than those not mentored yet. This implies that supportive supervision and mentoring are key determinants because experts would support, teach, discuss and control the system and practice of midwives at their own health facilities.

In this study, two midwives working in 1 shift were 89.9% times less likely to utilize partograph than eight midwives. Four midwives working in 1 shift were 89.5% times less likely to utilize partograph than eight midwives. A similar finding was observed in Rwanda [20],Cameroon [21],Nigeria-Calabar [27],Ghana-Kumasi [23]. This indicates that as the number of midwives is increased per shift, risk of under-utilization of partograph will be minimal. An increase in the number of staffs will make them not to be busy and can record all components of partograph.

This finding also showed that knowledge is a key determinant factor of partograph utilization. Midwives who were knowledgeable on partograph were 2 times more likely to utilize partograph than their counter parts. A similar study was observed in west Shoa zone Ethiopia [26], Bale Zone Ethiopia [25],Nigeria ile-ife [28],Ghana [23],Nigeria Calabar university [27], Rwanda [20],India [29] and Nigeria Enugu metropolis [30] .This enables them to understand what critical progress of labour will occur and decide on alternatives such as referral and caesarian section.

The strength of this study was that it gave equal chance to all participants since it uses simple random sampling method. However, inability to avoid Hawthorne effect totally, not testing reliability of the tool could be the limitation of this study.

## Conclusions and recommendations

More than half of the respondents utilized partograph during the progress of labour. About one third of midwives did not utilize the partograph having incomplete in their recordings. All public health institutions availd partograph in their laboring room, but didn’t utilize it according to WHO recommended standard. Working facility, supportive supervision, mentoring, training on partograph, and Knowledge of midwives on partograph showed positive association while number of midwives working per shift was negatively associated with utilization of partograph. Great commitment to utilize partograph; strengthened training system; regular supportive supervision; expertise mentoring; sustainable supply of partograph sheets; recruitment of midwives in acceptable number; a spirit of commitment for administrative bodies to improve maternal health and further study on predictors for utilization of partograph are recommended.

## Supplementary information

**Additional file 1: Annex I.** Revised WHO Partograph.

## Data Availability

The datasets generated and/or analyzed during the current study are available from the corresponding author on reasonable request.

## Abbreviations

AARHB: Addis Ababa Regional Health Bureau
CHEWs: Community Health Extension Workers
ETB: Ethiopian Birr
HC: Health Center
HEWS: Health Extension Workers
HFCP: Health Facility Core Process
MMR: Maternal Mortality Rate
MNH: Maternal and Neonatal Health
NGO: Non-Government Organization
PHCU: Primary Health Care Unit
RHB: Regional Health Bureau
SD: Standard Deviation
SPSS: Statistical Package for Social Sciences
UNICEF: United Nation Children’s Fund
WHO: World Health Organization
