# Supplementary material for: Utilization of Partograph and its associated factors among midwives working in public health institutions, Addis Ababa City Administration,Ethiopia,2017
Source: BMC Pregnancy Childbirth. 2020 Jan 21;20:49. doi: 10.1186/s12884-020-2734-4 (PMC6975085; doi:10.1186/s12884-020-2734-4)
Supplement: Supplementary file 1 — Additional file 1: Annex I. Revised WHO Partograph. [file 12884_2020_2734_MOESM1_ESM.docx]

Annex I: Revised WHO Partograph
